# Current status of transplantation in Brazil

**DOI:** 10.1590/S1516-31802010000100001

**Published:** 2010-01-07

**Affiliations:** I Associate professor, Department of Cardiopneumonology, Faculdade de Medicina da Universidade de São Paulo (FMUSP), São Paulo, Brazil.; II Director, Department of Kidney and Pancreas Transplantation, Santa Casa de Misericórdia de Porto Alegre, Porto Alegre, Brazil.

In 1964, kidney transplantation began in Brazil and, in 1968, heart, liver, intestine and pancreas transplantations were performed. Like elsewhere in the world, the disappointing results led to suspension of the transplant programs in the early 1970s, for all organs except kidneys.^1^ The discovery of cyclosporine in the 1970s, and its clinical application as immunosuppressive medication in early 1980, provided better results from kidney transplantation. Subsequently, the programs for heart (1984), liver (1985) and pancreas transplantation (1987) were reactivated and an early lung transplantation program was instituted in Brazil (1989).^1^

Regarding regulation, transplantation can be divided into three phases. The first, in which demand and allocation of organs was the responsibility of the transplantation centers, with no control from the Ministry of Health or other government bodies, extended from 1964 to 1987.^1^

The second phase started in 1987, with the publication by the Ministry of Health of the Integrated Plan for Chronic Renal Patient treatment (SIRC-TRANS), which was designed to set standards for the accreditation and functioning of kidney transplantation centers, and to determine the amounts for payment by the public health system.^2^ In the same year, in some states, government agencies and foundations took over responsibility for procurement and/or allocation of organs. The first organizations were the Immunogenetics and Organ Transplantation Program (PITO) in Rio de Janeiro,^3^ the São Paulo Interior Transplantation (SPIT) system^4^ and the Rio Grande do Sul Transplant Coordination service (RS-Tx).^5^ The Constitution of 1988 prohibited the sale of organs, and this was regulated by the transplantation law of 1992.^6^ In the same year, the Ministry of Health created the Integrated System for High Complexity Procedures (SIPAC), for kidney, liver, heart, lung and bone marrow transplantation. This established quality control mechanisms for transplant centers, accreditation for regulated transplantation services and set the amounts to be paid for these procedures.

The third phase began in 1998 with new legislation on transplantation and the creation of the National Transplant System (SNT) and the Centers for Notification, Procurement and Distribution of Organs (CNCDOs) in the states and Federal District. Controlling and funding all the processes of donation and transplantation became the responsibility of the federal government.^1^ Overall coordination of the SNT was assisted by Technical Advisory Groups (GTAs) and Technical Councils, with regard to specific organs and tissues, and the SNT was made responsible for transplant policy in this country. The CNCDOs controlled the logistics of donation and allocation in the states.^1^ In 1999, the National Center for Notification, Procurement and Distribution of Organs (CNNCDO) was created. It was established at the airport of Brasilia and was made responsible for the distribution of organs among all the states.^1^ In 2000, based on the Spanish model and at the request of the Brazilian Association of Organ Transplantation, the Transplantation Hospital Coordinator was created. This subsequently became known as the Intra-Hospital Committee for the Donation of Organs and Tissues for Transplantation (CIHDOTT), which was used in most states. São Paulo continued with the American model of using Organ Procurement Organizations (OPOs).^1^ Between 2000 and 2004, dozens of training courses were conducted for hospital transplantation coordinators in 18 states.^7^

With this organizational system, the donor rate in this country remained stable at around three per million population (pmp) between 1993 and 1998 and increased to 7.4 pmp between 1999 and 2004. However. from 2005 onwards, there was a progressive decrease in the donor rate, reaching 5.4 pmp in June 2007. This was caused by a number of factors, such as changes in the SNT that interrupted the courses. This led to a series of changes and improvements in transplantation policy that reversed this situation. Two years later (September 2009), the donor rate was 8.6 pmp, an increase of 54% over this period, thereby reaching the goal proposed for this year. However, the efforts in this area need to be redoubled in order to achieve the proposed donor rate targets of 10 pmp in 2010, 14 pmp in 2013 and 20 pmp in 2017.

The forecast for 2009, from the data obtained up to September, is that 4,130 kidney transplants (21.8 pmp) will be performed in Brazil, i.e. 31% of the 13,300 cases that enter the waiting list each year (70 pmp); 1,301 liver transplantations (6.9 pmp): 31% of the 4,160 needed (25 pmp); 193 heart transplantations (1.0 pmp): 18% of the 1,104 needed (6 pmp); 169 pancreas transplantations (0.9 pmp): 30% of the 570 needed (3 pmp); and 63 lung transplantations (0.3 pmp): about 4% of the 1,472 needed (8 pmp). The use of organs in Brazil is highly variable: exceeding 70% for kidneys and livers, around 15% for hearts and only 5% for lungs.^8-10^

Regarding the transplantation of corneas, which can also come from donors up to six hours after death, 13,052 transplants (71 pmp) are expected to be performed, i.e. 79% of the 16,560 needed (90 pmp). Some states, like São Paulo and Paraíba, have already been able to bring down the waiting list for corneas to zero.^8,9^

For organ transplantation to increase in Brazil, it is essential to improve the four pillars that support the donation process for transplantation: legislation, financing, organization and education. The legal measures include the implementation of a registration system for voluntary donors, and the prevention of any form of trade through greater control over transplants from unrelated living donors and prohibition of transplantation involving deceased donors who were non-resident aliens in the country. With regard to financial measures, adjustments to the funding available for organ procurement and transplantation need to be made, in order to include new medications like belatacept, rituximab and bortezomib as medicines supplied by the public system for transplantation patients, and to include payment by the public healthcare system for new diagnostic procedures such as C4d in biopsies, antigenemia or polymerase chain reaction (PCR) tests for diagnosing cytomegalovirus and quantitative measurement of viral load in cases of Epstein-Barr virus (EBV) and BK virus (BKV). Among the organizational measures deemed vital are training and motivation for intensive care physicians and neurologists, for diagnosing brain death and maintaining potential donors. Hospitals also need to be equipped through purchasing equipment to document brain death, training the hospital transplantation coordinators and having teams for organ procurement and transplantation available 24 hours a day in all states. Finally, educational policies are still needed, both for professionals and students in the healthcare field and for the population.
